# Variability in Cell Response of *Cronobacter sakazakii* after Mild-Heat Treatments and Its Impact on Food Safety

**DOI:** 10.3389/fmicb.2016.00535

**Published:** 2016-04-19

**Authors:** Julio Parra-Flores, Vijay Juneja, Gonzalo Garcia de Fernando, Juan Aguirre

**Affiliations:** ^1^Departamento de Nutrición y Salud Pública, Universidad del Bío-BíoChillán, Chile; ^2^Residue Chemistry and Predictive Microbiology Research Unit, Eastern Regional Research Center, Agricultural Research Service, United States Department of Agriculture, WyndmoorPA, USA; ^3^Laboratorio de Microbiología y Probióticos, Instituto de Nutrición y Tecnología de los Alimentos, Universidad de ChileSantiago, Chile; ^4^Departamento de Nutrición, Bromatología y Tecnología de los Alimentos, Facultad de Veterinaria, Universidad ComplutenseMadrid, Spain

**Keywords:** *Cronobacter sakazakii*, PIF/FUF, food safety, variability, risk assessment, heat treatment

## Abstract

*Cronobacter* spp. have been responsible for severe infections in infants associated with consumption of powdered infant formula and follow-up formulae. Despite several risk assessments described in published studies, few approaches have considered the tremendous variability in cell response that small micropopulations or single cells can have in infant formula during storage, preparation or post process/preparation before the feeding of infants. Stochastic approaches can better describe microbial single cell response than deterministic models as we prove in this study. A large variability of lag phase was observed in single cell and micropopulations of ≤50 cells. This variability increased as the heat shock increased and growth temperature decreased. Obviously, variability of growth of individual *Cronobacter sakazakii* cell is affected by inoculum size, growth temperature and the probability of cells able to grow at the conditions imposed by the experimental conditions should be taken into account, especially when errors in bottle-preparation practices, such as improper holding temperatures, or manipulation, may lead to growth of the pathogen to a critical cell level. The mean probability of illness from initial inoculum size of 1 cell was below 0.2 in all the cases and for inoculum size of 50 cells the mean probability of illness, in most of the cases, was above 0.7.

## Introduction

*Cronobacter* spp. are members of the family *Enterobacteriaceae* and can cause serious infection of all human age groups (Farmer et al., 1980; van Acker et al., 2001; Iversen and Forsythe, 2003; FAO/WHO, 2006; Holý and Forsythe, 2014), but infants of less than 1 year are at particular risk (Bowen and Braden, 2006; Pagotto and Farber, 2009) mainly the neonates, who are less than 4 weeks old (Yan et al., 2012). Severe symptoms related to *Cronobacter* spp., include necrotizing enterocolitis and meningitis (Simmons et al., 1989; van Acker et al., 2001; Himelright et al., 2002; FAO/WHO, 2004, 2006, 2008; O’Brien et al., 2009). The overall mortality rate caused by the microorganism is between 20 and 80% in infected infants (Lai, 2001; Hunter and Bean, 2013). The incidence of *Cronobacter* spp. infection among infants is relatively low (8.7 per 100 000 low birth weight neonates) (Cawthorn et al., 2008; Tóthová et al., 2011), however, it is assumed that the number of infections caused by Cronobacter is underreported (Tóthová et al., 2011).

Occasional contamination of powdered infant formula (PIF) and follow-up formulae (FUF) during manufacture is an important source of the microorganism’s occurrence in reconstituted product (FAO/WHO, 2008). However, *Cronobacter* spp. have been also detected in other foods (Friedemann, 2007) and environments such as food processing plants, hospital equipment and households (Kandhai et al., 2004; Guillaume-Gentil et al., 2005; Hurrell et al., 2009).

Powdered infant formula/follow-up formulae are not sterile (Farber, 2004; FAO/WHO, 2008). At least two outbreaks demonstrated a relationship between Cronobacter isolates from infected patients and the isolated cultured from unopened cans of PIF consumed by these same patients (Clark et al., 1990; Block et al., 2002). Although the levels of *Cronobacter* spp. are generally very low (<1 CFU/g) (Osaili and Forsythe, 2009), reconstituted infant formula is a good medium for growth (Parra et al., 2015). Furthermore, *Cronobacter* spp. may be recovered after, at least, 2.5 years from a dairy formula (Barron and Forsythe, 2007). When this bacterium contaminates one of these formulae, it may grow during preparation, cooling, storage, and holding of the reconstituted infant formula in bottles, *Cronobacter* spp. may grow resulting in an increased probability of illness. According to FAO/WHO (2006), the incidence rate among infants weighting less than 2500 g at birth was 8.7 per 100,000 infants in the United States of America during their 1st year of life. Other group of concern is the elderly who have experienced strokes that cause dysphagia and, therefore, may require reconstituted powdered supplements as part of their diet (FAO/WHO, 2006).

Recalls of infant formula contaminated with *Cronobacter sakazakii* have occurred in the United States, Europe, and other countries (Simmons et al., 1989; van Acker et al., 2001; Weir, 2002; Drudy et al., 2006; Schreck, 2010; Tomasulo, 2012; Herriman, 2015). This has resulted in increased efforts to implement appropriate strategies to reduce the health risks associated with the use of reconstituted infant formula and to provide guidance to risk-based management decisions (Reij et al., 2009; Pina-Pérez et al., 2014).

A microbial risk assessment (MRA) for *E. sakazakii* and other micro-organisms in PIF was developed in 2004 during a FAO/WHO expert meeting (FAO/WHO, 2004). This MRA was estimated in terms of relative risk, comparing different “what-if” scenarios regarding contamination levels and risk mitigation measures. During a second expert consultation in 2006, a more elaborate risk assessment model was presented, that allows comparing scenarios to a baseline scenario to be chosen by the user (FAO/WHO, 2006). In these MRAs risk estimates were made using the average response of microbial cells based on deterministic approaches, however, deterministic models are not effective in describing the behavior of small microbial populations or individual cells (Baranyi, 1998) since they ignore the proven variability between individual cells (Elfwing et al., 2004; Métris et al., 2005, 2008; Pin and Baranyi, 2006; Koutsoumanis, 2008; Niven et al., 2008; Dupont and Augustin, 2009; Aguirre et al., 2009; Cuevas-Muñoz et al., 2013; Lianou and Koutsoumanis, 2013; Aspridou and Koutsoumanis, 2015). Stochastic modeling approaches seem to solve the above problem since they are able to deal with more “realistic” food contamination events with few cells (Ross and McMeekin, 2003; Koutsoumanis, 2008; Koutsoumanis and Lianou, 2013; Alonso et al., 2014; Skandamis and Jeanson, 2015).

*Cronobacter* spp., especially the most virulent ones, may be present in reconstituted infant formula and possibly survive the mild heat stress associated with reconstitution because clinical strains appeared to be more thermotolerant than their environmental counterparts (Yan et al., 2012). On the other hand, as cross contamination can occur at any point, cells present in reconstituted infant formula may not be heat damaged. Despite the advice of FAO/WHO (2006) to use water at 70°C to reconstitute PIF, instructions for reconstitution may suggest use of water at temperatures as low as 40°C (Xu et al., 2015; Parra-Flores et al., 2015) due to the undesirable effects on the organoleptic, nutritional, and functional properties (Pina-Pérez et al., 2015) or risk of burns during preparation and feedings (Agence Francaise de Securite Sanitaire Des Aliments [AFSSA], 2005).

In both cases of contamination, it may lead to the PIF becoming unsafe, as untreated and mildly heat treated *Cronobacter* spp. present in the PIF may have the potential to recover and grow during the holding time. The assumption, in these cases, that all cells (treated and untreated cell survivors) will have the same lag phase and that all services will have the same dose per service (number of cells) can lead to unrealistic and inaccurate predictions, which is unlikely to be a sufficient basis for management decisions on the safety risk (European Commission [EC], 2002; Lammerding and Todd, 2006).

In contrast to extensive studies on individual lag for other microorganisms (Elfwing et al., 2004; Métris et al., 2005, 2008; Pin and Baranyi, 2006; Koutsoumanis, 2008; Niven et al., 2008; Aguirre et al., 2009; Cuevas-Muñoz et al., 2013; Aspridou and Koutsoumanis, 2015), few data are available on variability of single cell lag time of *Cronobacter* spp. (Miled et al., 2011; Xu et al., 2015).

In this study we compared both lag phases, estimated by deterministic and stochastic approaches of two inoculum sizes (individual single cell and fifty cells) of untreated or sublethal heat treated *C. sakazakii* at four storage and abuse temperatures. In addition, the effect of both approaches on the probability of illness was assessed.

## Materials and Methods

### Culture Preparation

*Cronobacter sakazakii* ATCC 29544 was used. The strain was kept frozen at -20°C in tryptic soy broth (TSB; Pronadisa, Madrid, Spain) supplemented with 20% glycerol. The strain was subcultured twice in sterile TSB at 37°C for 24 h to reach the stationary phase, with a concentration of ca. 10^9^ CFU/ml. Cells were harvested by centrifugation at 10,000×*g*, 15 min at 4°C in a Sorvall RC5B refrigerated centrifuge. The final pellets were resuspended in sterile saline solution (0.75% NaCl). Cell suspensions were then used to inoculate sterile TSB solution.

### Heat Shock

Cells were treated at 50° C during 5 and 10 min, based on the experiments described by Parra-Flores et al. (2015) and Xu et al. (2015).

To apply the heat treatments, the protocol described by Aguirre et al. (2009) was used with few modifications. Briefly, 10 tubes for each time of treatment containing 9.9 ml of sterile TSB were immersed in a temperature-controlled water baths (model TFB, Bunsen S.A., Madrid, Spain) set at the target temperature which was monitored using a thermocouple (Testo AG 720, Kirchzarten, Germany). Once tubes reached the target temperature, 100 μl of the bacterial suspension were inoculated. When the heat treatment was complete, at each sampling time (5 and 10 min), aliquots of 100 μl were removed and immediately mixed with 900 μl of cold sterile TSB in a tube immersed in an ice water bath. Surviving bacteria were properly diluted and plated on tryptic soy agar (TSA, Pronadisa, Spain) for counting. Initial inoculum size (non-heated cells) was also estimated to determine the degree of inactivation achieved.

Based on the methodology of Koutsoumanis (2008), from the above suspensions and untreated ones, a dilution containing around 100 cells (by previous plate counting) was plated in TSA to estimate the cell growth probability (Pg) of treated and untreated ones. Plates were incubated at 5, 10, 15, or 25°C for 30, 20, 10, and 5 days, respectively. Before storage, plates were covered with Parafilm (Parafilm ‘M’, American National Can, Greenwich, CT, USA) to avoid dehydration.

### Inoculum Size, Growth Rate, Growth Temperature, and Lag Phase

From the above two treated and untreated suspensions (50° C by 5 and 10 min of exposure), serial-dilutions in TSB were prepared in order to obtain several inoculum sizes from 10^6^ to 10^1^ cells/ml as described in Aguirre et al. (2013).

The specific growth rate (μ_max_) was estimated in TSB at 5, 10, 15, and 25°C from turbidity growth curves in three replicate experiments using an automated spectrophotometer (Bioscreen C, Labsystems, Helsinki, Finland) kept in a controlled temperature room as described in several studies (Guillier et al., 2005; Métris et al., 2005; Aguirre et al., 2013).

Briefly, from the above mentioned inoculum sizes, 20 replicate samples (350 ml) from each dilution were inoculated into wells of Bioscreen microplates (honeycomb plates, Thermo Fisher Scientific, Basingstoke, UK). The plates were loaded into the Bioscreen C at incubation temperatures of 5, 10, 15, or 25°C. After shaking at medium intensity for 10 s, turbidity measurements were determined using a wide band filter at 420 to 580 nm at 15-min intervals. Plates were incubated for enough time to reach stationary phase in the most diluted samples [for up to 1 month or enough time to observed optical density (OD) above 0.35]. The reading chamber of the Bioscreen was pre-heated to a set-point temperature 1 day before the experiment to allow equilibration.

Dilutions of each initial inoculum were plated by spreading onto TSA, then they were incubated at 5, 10, 15, or 25°C in a controlled incubator for the same time as the Bioscreen experiment’s and finally colonies were counted. Using the Bioscreen device, the time to detection (Td), defined as the time required to reach an absorbance of 0.20 (Aguirre et al., 2012, 2013), was obtained from each well, and a mean value was calculated for each dilution. μ_max_ was estimated from the reciprocal of the absolute value of the regression slope of the Td versus Ln (N), where N is the initial number of cells. The experiment was carried out at least three times.

To estimate the lag time (λ) in TSB from the solutions mentioned above (heat shocked at 50°C during 5 and 10 min and untreated ones), the same protocol described by Aguirre et al., 2013 was followed using the Bioscreen C equipment with few modifications. To do this, 350 μl from the dilution expecting that contain 10^1^ and 10^0^ CFU/ml (100 samples per dilution) were transferred to the two microplates of the Bioscreen (100 wells per dilution) and incubated at 5, 10, 15, or 25°C, kept in at a controlled room temperature. The increase in OD was tracked by measuring it in the wavelength range from 420 to 580 nm using the Bioscreen C every 30 min for up to 30, 25, 15, and 10 days for 5, 10, 15, or 25°C, respectively. Cultures were shaken for 10 s at medium intensity before OD was measured.

Lag times were estimated using the detection time (Td), defined as the time required for the OD in the wavelength range between 420 and 580 nm to reach 0.2 units, which corresponds to an average concentration of 1.57^∗^ 10^7^ CFU/well obtained by OD calibration curve (data not shown) as described in previous studies (Métris et al., 2005, 2006; Aguirre et al., 2013). More specifically, lag times were estimated based on the following equation (Baranyi and Pin, 1999):

$$Lag=T_{d}-(\frac{Ln\,\;(Nd)-Ln\,\;(N_{0})}{\mu })\,\;\;\;\;\;\;\;\;\;\;\;\;\;\;\;\;(1)$$

where Nd is the bacterial number (CFU) at Td, N_0_ the initial number of cells (CFU), and μ (h^-1^) is the specific growth rate determined from the growth curve obtained under the experimental conditions described above.

To estimate the initial average number of cells (N_0_) per well that were able to grow at each growth temperature, 350 μl from each dilution were mixed with molten TSA in plates and incubated at 5, 10, 15, or 25°C in a controlled incubator for the same time as the lag time experiments and then colonies were counted. Approximately 20 plates were counted for each dilution and treatment. To be considered, Guillier et al. (2005) stated that if 35% of samples (microplates) show growth, this should not significantly affect individual cell lag phase distributions because at least 80% of samples contain one cell, according to the Poisson distribution function (Francois et al., 2003). Finally, number of cells per well was assessed based on the number of positives as described in Aguirre et al. (2012) and each experiment was replicated two or three times to obtain at least 80 individual cell lag times. A similar protocol was used to estimate the kinetic parameters of 50 cells per well.

### Data Analysis and Modeling

The data of λ and μ_max_ were fitted to various distributions using the @Risk 4.5 for Excel software (Palisade Corporation, Newfield, NY, USA). The goodness of fit was compared using three different methods: *X*^2^, Anderson–Darling (A–D) and Kolmogorov–Smirnov (K–S). The best-fitted distributions based on the mentioned criteria were further introduced into an exponential model with lag to describe the growth of individual treated and untreated cells using Monte Carlo simulations as described by Koutsoumanis and Lianou (2013):

$$N_{t}=\left(N_{0}-N_{g}\right)+\overset{N_{g}}{\underset{1}{\Sigma }}\left\{\begin{array}{cc} 1 & for\,\;t\leq \lambda_{t}\\ e^{\mu \,\max t\left(t-\lambda_{t}\right)} & for\,\;t>\lambda_{t} \end{array}\right\}$$

where N_t_ is the total number of cells in a population at time t, N_0_ is the initial number of cells in the population at *t* = 0, Ng∼Binomial (N_0_, Pg) is the initial number of cells in the population at *t* = 0 that are able to grow and form a colony, Pg is the mean probability of growth at each condition (heat shock and growth temperature) determined as described by Koutsoumanis (2008), μ_max_ and λ are introduced as probability distributions from the best fit, respectively, of a microcolony originating from an untreated and a treated single cell, respectively. The output of the model was assessed for N_0_ of 1 and 50 cells using Monte Carlo simulation with 10,000 iterations and with a uniform distribution for t [t∼Uniform (0, tn)], with tn 250, 600 and 900 h for each temperature of cells untreated and heat treated at 50°C for 5 or 10 min, respectively.

The above approach takes into account the heterogeneity (variability) in the growth dynamics of single cells by introducing the kinetic parameters in the model as probability distributions using Monte Carlo simulation. In addition, this approach predicts the probability of growth of individual cells as a function of prior heat treatment.

### Growth in PIF

To test the applicability of the model in commercial PIF, packages of infant product from the same batch were bought locally. Before use, they were checked for absence of anaerobic and aerobic microorganisms, including *Cronobacter* spp, based on the method of Chap et al. (2009). The PIF was reconstituted using sterile water at 50°C and 900 ml of the reconstituted milk were distributed in 100 Eppendorf tubes for each growth temperature (5, 10, 15, and 25°C). Once stabilized the temperature, tubes were inoculated with 100 μl of 1 or 50 *Cronobacter* cells that survived heat treatment at 50°C for 5 or 10 min or that were not subjected to prior heat treatment. Tubes were incubated at 5, 10, 15, and 25°C for up to 1 month and everyday duplicate samples were mixed in molten TSA in plates and incubated at 5, 10, 15, and 25°C.

The obtained growth curves were then fitted to the deterministic primary model of Baranyi and Roberts (1994) for the estimation of λ and μ_max_. As suggested by Koutsoumanis and Lianou (2013), in order to describe the abrupt transition from the lag to the exponential phase characterizing the observed growth, the values of the parameters *m* and *n* of the model were fixed to 0 and 20, respectively.

### Probability of Illness of Infants by *Cronobacter sakazakii* from Consumption of PIF

Through the consideration of the storage stages between preparation of the formula and feeding of the infant, we used the Risk Assessment Model for *Enterobacter sakazakii* in PIF designed by FAO/WHO (2006) although including the probability distribution provided by our data as an input of the model, hence the risk characterization provides the level of contamination, the ingested dose, and the probability of illness resulting from feeding PIF.

To include the uncertainty in our results and since infants can take different amounts of milk, we fitted a distribution to the weight of the infant and the expected consumptions provided by FAO/WHO (2006) which are shown in **Table 1.**

**Table 1:** Infant group definitions presents as options in the risk assessment model (modified from FAO/WHO, 2006).

Infant group	Definition	Weight (g)	Daily intake (ml/kg/day)	ml/day
Extremely low birth weight	Birth weight <1000g	800	150	120
Very low birth weight	Birth weight <1500g	1250	200	250
Low birth weight	Birth weight <2500g	2000	200	400
Premature neonate	Prior to 37 complete weeks	2250	150	334
Term non-LBW Neonate	0 to 28 days of age	3600	150	540
Young infant	29 days to 6 months of age	5000	150	750
Older infant	6 to 12 months of age	9000	55.55	500


To estimate the probability of illness (P*_ill_*), the exponential dose-response model was used (Haas et al., 1999).

$$P_{ill}=1-\exp^{-rdc}$$

where *r* is the exponential dose-response parameter and *dc* is the dose at consumption that results from an initial contamination level of 1 or 50 cells of *Cronobacter* spp per serving.

As mentioned by FAO/WHO (2006), there are no data currently available to estimate the parameter *r*, however, in the report they proposed six options for *r*, ranging from 1^∗^10^-5^ to 1^∗^10^-10^. In our approach, a Uniform distribution was used with these values. To estimate *dc*, the volume of consumption and the concentration of *Cronobacter* spp. at the time of consumption have to be considered. A Normal distribution (413.39; 206.61) was fitted to the estimated milk consumption, which was obtained by the product between weight of the infant and the daily intake shown in **Table 1.**

To estimate the concentration of *Cronobacter* at the time of consumption, two approaches (probabilistic and deterministic) were assessed based on our data, considering the time to reach a hypothetical infected dose of 1000 CFU (Iversen and Forsythe, 2004; Mittal et al., 2009) with an initial concentration of 1 or 50 untreated cells or heat treated at 50°C for 5 or 10 min and the concentration reached after 50 h of incubation at 15°C for a single cell survivors to heat treatment at 50°C for 5 or 10 min.

Finally, to have an idea of the probability of illness in real a situation, Eq. 3 was multiply by the prevalence of *Cronobacter* in PIF obtained from the literature (Iversen and Forsythe, 2004; FAO/WHO, 2008; Chap et al., 2009; Siqueira et al., 2013), from 3 to 30%. To do this, we assumed a Uniform distribution (0.03, 0.3).

## Results

Experimentally obtained lag phase and specific growth rate values are shown in **Table 2.** As expected, the higher the growth temperature, the shorter the lag phase was, reflecting the known mesophilic character of this species (Nazarowec-White and Farber, 1997). At the same growth temperature, the lag phase increased as the time of exposure to heat treatment increased with untreated cells showing the shortest lag phases. For example, at 25°C, lag phase was 3.9 h for an untreated single cell whereas it was 424 h for a single cell that survived at 50°C for 10 min. In the case of the inoculum size of 50 cells, the shorter lag was 1.75 h and the higher was 272 h in the same conditions as those of 1 cell.

**Table 2:** Kinetic parameter (lag and umax) of 1 and 50 cells of *Cronobacter sakazakii* growth of cells able to growth at three different temperatures in tryptic soy broth (TSB).

Cell (s)	Growth (°C) temperature	treat (min)	mean lag (h)	SD (lag h)	Growth rate (LnN) h^-1^
1	5	0	84.6	10.61	0.035
		5	285.6	78.04	0.037
		10	424.0	101.19	0.038
	10	0	32.0	5.28	0.097
		5	108.5	33.24	0.098
		10	198.5	51.00	0.097
	15	0	14.9	2.87	0.278
		5	49.4	16.44	0.275
		10	97.8	27.86	0.279
	25	0	3.9	1.26	1.134
		5	17.5	7.02	1.135
		10	36.6	12.89	1.134
50	5	0	45.8	8.49	0.039
		5	177	48.24	0.038
		10	272.6	77.22	0.038
	10	0	15.9	4.12	0.098
		5	59.9	26.11	0.098
		10	116	42.51	0.099
	15	0	5.08	1.92	0.280
		5	27.38	11.62	0.279
		10	58.58	20.01	0.278
	25	0	1.75	0.87	1.139
		5	10.08	4.38	1.141
		10	20.35	8.03	1.143


Inoculum size also affected the lag phase (**Table 2**). Lag phase of single cells were longer on average than lag phase for samples that contained 50 cells. In contrast, growth rates did not show substantial differences between treated and untreated cells at the same growth temperature. In addition, the specific growth rate was not affected significantly by inoculum size. However, as expected, temperature did affect the growth rate; the higher the temperature the higher the growth rate. For example, the highest mean growth rate (1.134 h^-1^) was obtained with cells grown at 25°C, and the lowest mean growth rate (0.035 h^-1^) was observed with cells grown at 5°C, a decrease of approximately 97%.

**FIGURE 1:**
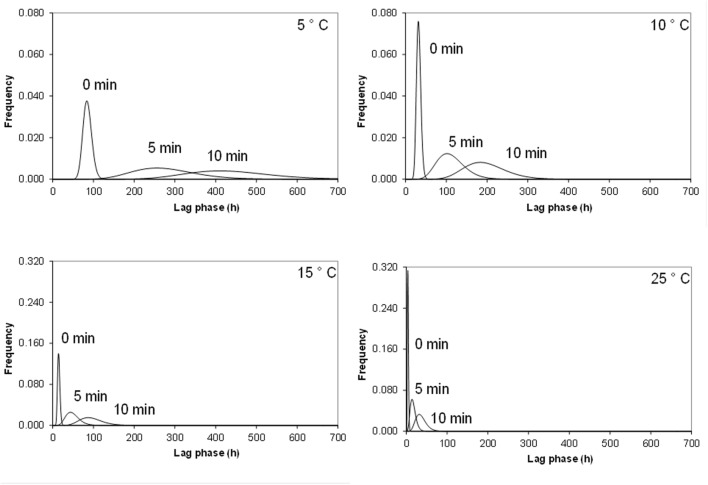
**Frequency distribution of individual *Cronobacter sakazakii* lag phases fitted by gamma distribution at different growth temperatures after 50°C heat shock by 0, 5, and 10 min**.

**Figure 1** shows the fitted Gamma distributions for lag phase of single cells of *Cronobacter sakazakii* as a function of temperature and heat treatments. For the same growth temperature, the variability of the lag phase increased as the severity of heat shock increased. For example, at 5°C, the lag phase of the untreated samples ranged from 45 to 125 h whereas it ranged from 80 to 420 h for cells heat shocked at 50°C for 5 min and from 176 to 650 h for cells heat shocked at 50°C for 10 min. As the growth temperature increased, the distributions for lag phase became less variable (**Figure 1**). Similar results were obtained for the higher inoculum size (i.e., 50 cells) although the distributions were narrower; reflecting the smaller standard deviations than those of 1 cells (data not shown).

**FIGURE 2:**
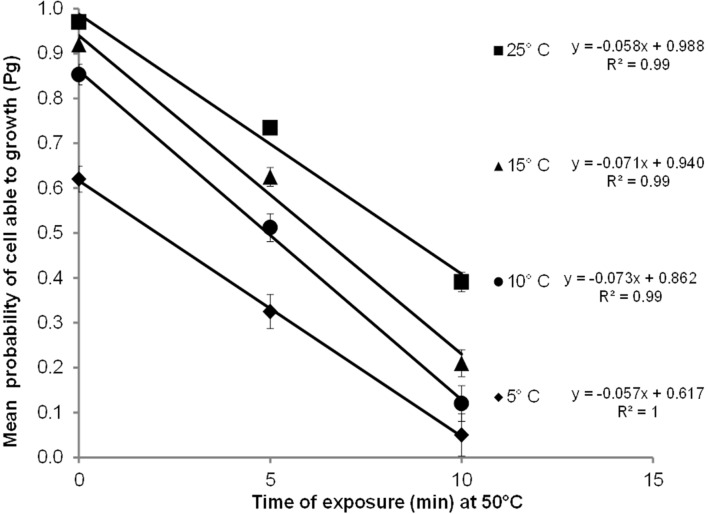
**Probability of growth (Pg) of *C. sakazakii* as a function of exposure time at 50°C by 0, 5, and 10 min and growth temperatures of 5, 10, 15, and 25°C.** The continuous black lines, for which equations are shown in the figure, show the relationships found in our experiments.

**Figure 2** shows the mean probability of cell able to growth (P_g_) as a function of the time of heat treatment and temperature. It was observed that as the heat treatment increased, P_g_ decreased; in addition, the higher the temperature, the higher was the Pg, indicating a significant variation in the number cells with growth ability. For example, at 25°C, the Pg was 0.05 for cells that survived heating at 50°C for 10 min, whereas the Pg was 0.97 for cells treated for 10 min at 50°C.

We found an explicit linear relationship (**Figure 2**) between Pg and the heat treatment at the three different growth temperatures with a high coefficient of determination (*R*^2^ ≥ 0.99). We also observed variability in the Pg within the same time of treatment. This variability increased when the heat treatment increased and the growth temperature decreased (presented in the **Figure 2** as bar of standard deviation). However, the standard deviation come from 10 observations, that the authors consider not enough to result in robust results and to affirm that the probability of growth is more variable when growth is less probable, hence, we continued working with the mean Pg, but this observation can be important for further research.

**Table 3** describes the estimated parameters of the distribution fitted to the experimental data and the variables used in Monte Carlo simulations. The best fitted distribution (according to *X*^2^, A–D, or K–S test, data not shown) to the experimental lag phases and specific growth rate were gamma and normal, respectively.

**Table 3:** Incubation temperature, duration of heat treatments, fitted distribution and parameters used to simulate growth of untreated and heat treated cells of a single cell of *C. sakazakii.*

		Distribution of parameter	
		
Growth temperature (°C)	Treat (min)	Lag (h)	Specific m (h^-1^)
5	0	Gamma [7.8; 3.8; Shift(55)]	Normal [0.03; 0.01; Shift(0.005)]
	5	Gamma [11.438; 23.076; Shift(21.629)]	Normal [0.03; 0.05; Shift(0.007)]
	10	Gamma [11.028; 30.471; Shift(87.929)]	Normal [0.03; 0.06; Shift(0.008)]
10	0	Gamma [2.9; 3.1; Shift(23)]	Normal [0.092; 0.01; Shift(0.005)]
	5	Gamma [8.5; 11.4; Shift(11.629)]	Normal [0.0912; 0.05; Shift(0.007)]
	10	Gamma [12.9; 14.2; Shift(15.29)]	Normal [0.091; 0.05; Shift(0.006)]
10	0	Gamma [1.7; 2.2; Shift(11.14)]	Normal [0.25; 0.02; Shift(0.028)]
	5	Gamma [6.4; 6.5; Shift(7.84)]	Normal [0.25; 0.02; Shift(0.025)]
	10	Gamma [9.8; 8.9; Shift(10.41)]	Normal [0.25; 0.02; Shift(0.029)]
25	0	Gamma [1.1; 1.2; Shift(2.54)]	Normal [1.13; 0.05; Shift(0.004)]
	5	Gamma [3.6; 3.7; Shift(4.17)]	Normal [1.133; 0.06; Shift(0.002)]
	10	Gamma [5.7; 5.4; Shift(5.84)]	Normal [1.133; 0.03; Shift(0.001)]


**FIGURE 3:**
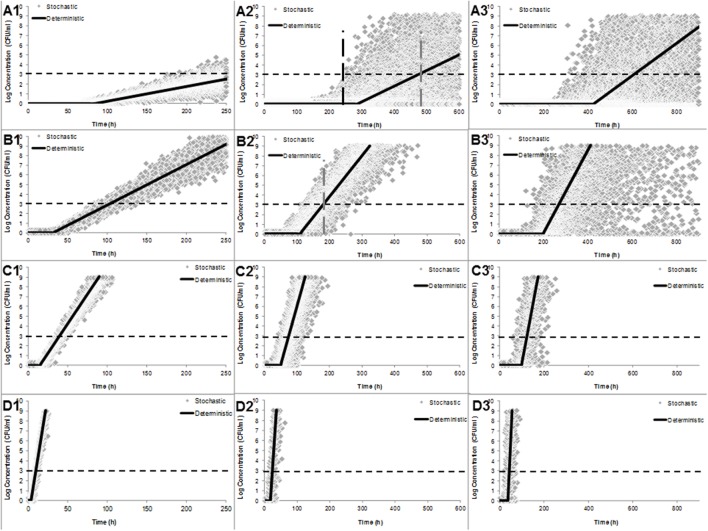
**Simulation output of stochastic (gray diamonds) and deterministic (continuos black lines) growth models of an individual *C. sakazakii* at 5 **(A)**, 10 **(B)**, 15 **(C)**, and 25 °C **(D)** and treated with 50°C by 0, 5, and 10 min (numbers 1, 2, and 3, respectively).** Discontinuous vertical lines **(A2)** represent the time at which deterministic and stochastic models reach the concentration of 10^3^ CFU/ml. Stochastic predictions for the growth of a single cell using Monte Carlo simulation with 10,000 iterations.

**Figure 3** shows the fitted distributions together with the density data for λ and μ_max_ of one cell based on the stochastic growth model (Eq. 2) in TSB at 5 (A), 10 (B), 15 (C), and 25°C (D) and simulated using Monte Carlo approach with 10.000 iterations and the deterministic growth model of Baranyi and Roberts (1994) fitted to data of *C. sakazakii* in PIF. From the stochastic approach it can be observed that the higher the heat treatment, the higher the dispersion of the data [from right (3) to the left (1)]. This dispersion decreased as the growth temperature increased [from 5°C (A) to 25°C (D], indeed deterministic and stochastic approaches were similar at 25°C (**Figures 3D1–D3**). In addition, it can be observed that the tendency of the fitted model is situated in the average of the stochastic outputs. For example, at 5°C, the stochastic growth model predicted that the dangerous dose level of 1000 CFU/ml was reached at 250 min (discontinuous black line **Figure 3A2**), while the deterministic approach indicated that this concentrations was reached after 490 min (discontinuous gray line **Figure 3A2**) from a single survivor that was heat shocked at 50°C for 5 min and growing at 5°C. A similar tendency was observed in the simulations of an inoculum size of 50 cells (results not shown). In addition, it can be observed in the vertical discontinuous gray line in **Figure 3B2**, the differences in the concentrations at the time 190 min (when deterministic model reaches 1000 CFU/ml), the stochastic growth model at the same time can have concentrations ranging from 0 to 10^7^ CFU/ml.

The comparison between stochastic and deterministic approaches showed a relevant difference between the probabilities of illness determined by both approaches. For example, the deterministic approach predicted (using Eq. 3) a probability of illness of 0.144 for a dose of 1000 CFU/ml, in contrast, the probabilistic approach predicts a probability of illness ranging from 0 to 0.285 for untreated cells, 0 to 0.071 for cell treated for 5 min at 50°C and from 0 to 0.002 (95% of the cases) when deterministic growth model reached 1000 CFU/ml (Concentrations are taken from **Figures 3C1–C**_3_, respectively). Note that for every case the time to reach 1000 CFU/h was different.

**FIGURE 4:**
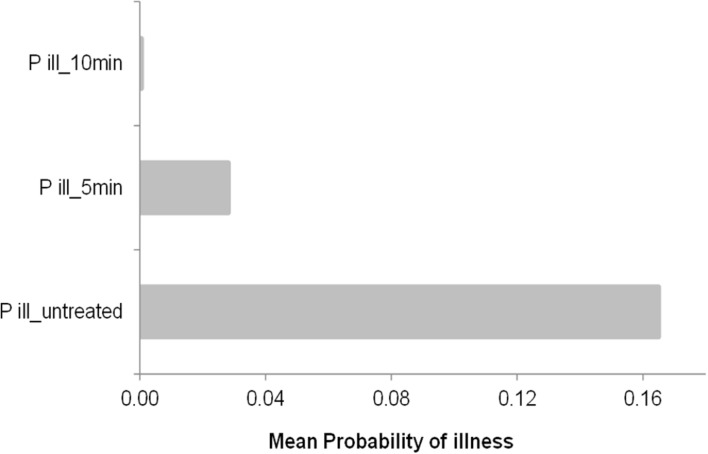
**Mean probability of illness by consumption of PIF contaminated with a concentration of cells (taken from **Figures 3C1–C3**, stochastic approach at 50 h of incubation at 15°C of a single cell survivor to 50°C for 5 and 10 min).** The control is also showed as untreated cells and the prevalence is included as a uniform distribution between 3 and 30%.

As expected the previous finding was affected by growth conditions and previous history of the cells (heat treatment intensity). The higher the previous heat treatment the lower the probability of illness, indeed the predicted mean probability of illness in this conditions was close to 0 (0.0006) as shows **Figure 4**, in contrast, untreated cells showed the higher mean probability of illness (0.165), while heat treated cells for 5 min had a mean probability of 0.003. These estimation are affected directly by the concentration of cells at the time of consumption, in this example, after 50 h of keeping the PIF at 15°C, the stochastic growth model of untreated cells (**Figure 3C1–C**_3_) predicted concentration ranged from 2282 to 32205 cells/ml (95% CI) while heat treated cells by 50°C for 10 min there were no growth (1 cell) to 3 cells/ml (95% CI), in the other hand, at 5 min of heat treatment the concentration observed was 1 to 873 cells/ml (95% CI).

In contrast, deterministic growth model predicted a concentration after 50 h at 15°C of 16384 cells/ml for untreated cells, 2 and 1 cells/ml for cells heat treated at 50°C for 5 and 10 min, respectively. These concentrations resulted in probability of illness of 0.165, 0.0007 and 0.0003, respectively.

**FIGURE 5:**
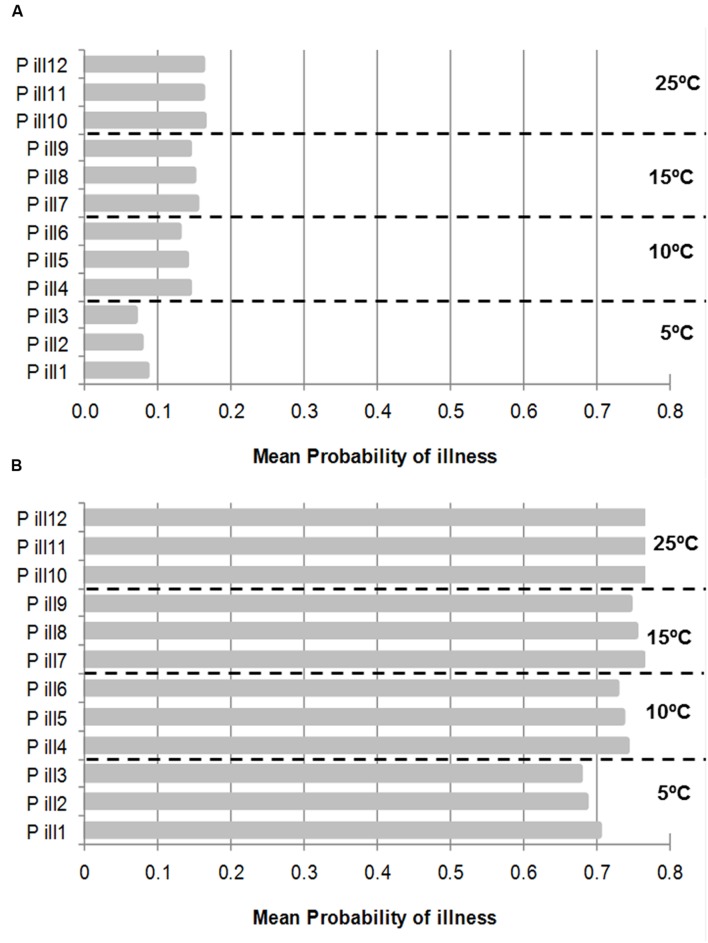
**Mean Probability of illness by consumption of PIF contaminated with an initial inoculum of 1 cell **(A)** and 50 **(B)** cells and growth at four different temperatures.** Each temperature has three probability of illness according to cells treated with 50°C by 0 (P ill 1), 5 min (P ill 2), and 10 min (P ill 3) and so on, respectively. The concentration to estimate the probabilities were taken from stochastic growth model using the cut-off point when the deterministic growth model reached 1000 CFU/ml (See **Figure 3**).

**Figure 5** shows the mean probability of illness by consumption of PIF contaminated with an initial inoculum of 1 (**Figure 5A**) and 50 (**Figure 5B**) cells growing at four different temperatures with the input of the stochastic growth model at the concentrations when the deterministic model reached 1000 CF/ml (**Figure 3**). It can be observed that the effect of the temperature and the inoculum size in the probability of illness. As expected the higher inoculum size and growth temperature were, the higher the mean probability of illness. The probability of illness from initial inoculum size of 1 cell were below 0.2 in all the cases and for inoculum size of 50 cells the probability of illness were, in most of the cases, above 0.7; the probability of illness was 0.68 and 0.69 by consumption of PIF with cell survivor to 50°C by 5 and 10 min, respectively (**Figure 5B**).

In contrast, it can be observed that there were no differences among untreated or heat treated probabilities of illness for the two times considered (5 and 10 min) within the same growth temperature and inoculum size. To note that, to estimate the probability of illness it was used the output of the stochastic growth model but when the deterministic model reached 1000 CFU/ml, in this point of cut-off, the distribution of cells of treated and untreated cells were similar (See **Figure 3**).

## Discussion

A major goal of scientists, industry, public health, and regulatory authorities is to control pathogenic microorganisms and improve food products hygiene and safety within a country and internationally (Sofos and Geomaras, 2010). However, in reality, it would be impossible to measure all parameters and conditions under which food pathogens, such as *Cronobacter* spp. can growth. Deterministic models for the behavior of microorganism are not effective in describing the behavior of small microbial populations or individual cells (Baranyi, 1998), since they ignore the observed variability between individual cells (Nauta, 2000; Koutsoumanis, 2008; Niven et al., 2008; Métris et al., 2008; Aguirre et al., 2009; Lianou and Koutsoumanis, 2011; Cuevas-Muñoz et al., 2013; Aspridou and Koutsoumanis, 2015). Stochastic modeling approaches seem to solve the above problem since they are able to deal with more “realistic” food contamination events with few cells (Koutsoumanis, 2008).

In this study we compare both lag phases, estimated by deterministic and stochastic approaches of two inoculum sizes (individual single cell and fifty cells) of untreated or sublethally heat treated *C. sakazakii* at four temperatures and the effect of both approaches on the probability of illness was assessed.

We observed considerable variability in the lag phase in our experiments (**Figure 1**), with a remarkable influence of the previous heat shock, the inoculum size and the growth temperatures (**Table 2**); in addition, we observed an effect of the heat shock and growth temperature on the probability (Pg) of cells able to growth (**Figure 2**). Both findings are in agreement with similar results in previous studies (Koutsoumanis, 2008; Métris et al., 2008; Aguirre et al., 2011, 2013; Lianou and Koutsoumanis, 2011). Miled et al. (2011) confirmed that the variability of individual cell lag times has a major impact on the growth of *Cronobacter*; however, the microorganism was subjected to dry stress, rather than mild heating. Recently, Xu et al. (2015) also observed that lag phase of individual *C. turicensis* cells was variable after mild heat treatment. They stated that an increase in the stress on cells resulted in increases in lag variability, as mentioned also by other authors (Stephens et al., 1997; Smelt et al., 2002; Métris et al., 2006; Rasch et al., 2007).

Generally, the specific growth rate in TSB (**Table 2**) in this study is in agreement with previous studies (Nazarowec-White and Farber, 1997; Iversen et al., 2004; Kandhai et al., 2004; Ghassem et al., 2011; Miled et al., 2011; Xu et al., 2015), the higher the growth temperature, the higher the growth rate.

In the published scientific literature, many researchers have characterized and explained the reason of kinetic variability from a biological point of view. Since this aspect is beyond the scope of this study, we direct the readers to different references for further information on the explanation of biological variability (Nauta, 2000; Robinson et al., 2001; Korobkova et al., 2004; Stewart et al., 2005; Avery, 2006; Lianou et al., 2006; Koutsoumanis and Lianou, 2013; Jackson et al., 2014; Huertas et al., 2015). However, we point out the information provided by Pin and Baranyi, (2006) who mentioned the presence of fast-growing cells and their subsequent subpopulations which have a dominant effect on the growth of the total culture (Pin and Baranyi, 2006), denoting the presence of different types of subpopulations, in this case, sensitive, less sensitive and resistant to environmental stressor, which can be supported by the several D values and its variation reported in the literature (Huertas et al., 2015). The presence of different types of cells will affect to the adaptation to the environmental condition, in the recovery and the ability to initiate growth. Also, it is important to mention that, not all cells within a population have the same probability to grow which is affected by the inoculum size (Koutsoumanis, 2008) and the growth temperature (Aguirre et al., 2013), which are expressed as genotype (Noma et al., 2006) or non-genotypic differences (den Besten et al., 2006), which is one of the biggest assumptions used in predictive microbiology models when survivors are counted at optimum growth conditions rather than at the temperature of the “real growth conditions”, which is demonstrated here. Although other factors may have a large impact on the exposure to *Cronobacter* spp., the initial level of the micro-organism in PIF is one of the key to its impact on public health and thus insight in this level is important for governmental risk managers as well as for PIF manufacturers (Reij et al., 2009).

We found that a gamma distribution described well the distribution of lag times whereas a normal distribution described well the distribution of μ_max_ for both heat stressed and unstressed *C. sakazakii* cells (**Table 3**). These findings are in agreement with those reported by others authors (Métris et al., 2008; Aguirre et al., 2013; Koutsoumanis and Lianou, 2013; Xu et al., 2015). In conditions of very low contamination, individual cell variability can have an important impact on pathogen growth (Guillier and Augustin, 2006). Knowing how their long term presence in PIF, and subsequent stress, affect the variability of single-cell lag times is important in assessing the risk of cell recovery and growth in reconstituted milk, where low numbers of stressed cells of pathogenic bacteria may be distributed among PIF samples (Miled et al., 2011) or may enter into the infant formula post preparation, during the manipulation or storage (Reich et al., 2010; Kucerova et al., 2011; Parra et al., 2015). Moreover, the heterogeneous distribution of *Cronobacter* cells in PIF makes this even more the case. In a real situation, a distribution of the inoculum size can be observed in a batch of food, which markedly influences public health risk. This heterogeneity can be due to the structural heterogeneity of the food matrix, incomplete mixing, incidental (post-processing) contamination, and/or localized microbial growth (Jongenburger et al., 2011). It is critical to take into account variability in microbial response because the credibility of a microbial risk assessment is based on its ability to consider the variability and uncertainty of each parameter involved in estimating final risk (Delignette-Muller and Rosso, 2000). The presence of a few atypical cells with short lag phase can unexpectedly shorten population lag time (Baranyi, 2002), which may shorten food shelf-life or, if pathogens are present in the food, increase the health risk to consumers.

The probabilistic approach proposed by Koutsoumanis and Lianou (2013) and replicated in the present study (**Figure 3**) is a stochastic growth curve in which the number of cells in the population at any time is a probability distribution based on a Monte Carlo simulation for describing the variability of parameters. For example, the number of cells in a microcolony generated from a single untreated cell after 100 h of growth at 10°C (**Figure 3B1**) can be either 155 (1st percentile) or 5252 (99th percentile); in contrast the deterministic model predicts a number of cell in average of 777, or from a heat shocked single cell (50°C for 5 min, **Figure 3B2**) growth at 10°C, the number of cells can be either 1 (1st percentile) or 76 (99th percentile), while the deterministic model predict no growth (1 cell with no duplication yet). The above variation in concentration of *Cronobacter* spp. at the time of consumption can affect the response of the host, however, in the case of this pathogen several aspects need more work to establish a proper dose-response model for premature newborns.

Here, it is easy to understand and visualize the impact of the variability of the kinetic parameters in the estimation of the concentration at certain time of growth and the impact between stochastic and deterministic approaches in our estimations, indeed, if we graph an imaginary line (**Figure 3A2**) we can observe that after 250 min the stochastic model predicted a concentration of 1000 CFU/ml while in the deterministic model, this concentration is reached after 490 min from a single cell growth sublethaly injured at 50°C for 5 min and grown at 10°C. Hence, the impact of individual cell variability on the growth of *Cronobacter* as affected by inoculum size, growth temperature, and the probability of growth should be taken into account, especially when errors in feeding bottle-preparation practices, such as improper holding temperatures, may lead to growth of the pathogen to a critical cell level (Miled et al., 2011). In addition, to better estimate risk (Aguirre et al., 2013) the above variability should be used in a dose response model, in contrast to providing the mean average probability of illness (**Figures 4** and **5**), however, care should be taken with the effect of the inoculum size, because even the best studied host–pathogen systems, the exact relation between the inoculum size and the probability of disease is unclear (Cornforth et al., 2015).

Current manufacturing processes are not capable of producing a sterile PIF (Kent et al., 2015), in addition, intrinsic contamination of PIF can occur at any stage during manufacture at the factory before distribution of product for retail; also, extrinsic contamination of product can occur after the factory container is first opened by the user; at any stage of reconstitution through the use of contaminated water, utensils, work surfaces; at the time of feeding (e.g., using contaminated feeding bottles or enteral tubing with existing biofilm); or because of inappropriate storage conditions (e.g., poor refrigeration or storage for too long at room temperature) (Kalyantanda et al., 2015).

In this study, we established the importance of assessing the impact of some factors (heat treatment, inoculum size, growth temperature) on the variability response of *Cronobacter* and its impact in the probability of illness by PIF consumption contaminated with it, however, several aspects need more work and scrutiny before being conclusive and able to us to performance a sensitivity analysis (Poschet et al., 2004; Membré et al., 2008; Ellouze et al., 2010). Additionally, the stress response factors identified previously in *Cronobacter* species, such as heat-shock, cold-stresses, survival in dry conditions, water activity (aw), and pH need to be re-assessed using novel approaches that are currently under development (Yan and Fanning, 2015).

## Conclusion

Extensive variation of lag phase was observed for *C. sakazakii*. The inoculum size also affected the lag phase, as the inoculum size decreased the mean lag phase and its variability increased. The μ_max_ was primarily affected by growth conditions and not by inoculum size. Results of this study highlight the risks associated with mean estimation rather stochastic approaches. Furthermore, the information provided here demonstrates that the effect of the growth environment and previous stress on the variability of the kinetic behavior of the microorganism survivors to a treatment is not negligible, and should, therefore, be characterized and taken into account in the development of stochastic approaches utilized in predictive microbiology and microbial risk assessment for food safety plans.

## Author Contributions

JP-F: He is an expert in *Cronobacter* spp., he was involved dose response model assess and risk assessment. VJ: Expert in food safety and predictive microbiology, he support the growth model approach. GF: Expert in food microbiology and predict microbiology, he was involved in experimental designing. JA: Risk assessment and predictive microbiology approach in this work and data processing.

## Conflict of Interest Statement

The authors declare that the research was conducted in the absence of any commercial or financial relationships that could be construed as a potential conflict of interest.
